# Mandibular Oral Tori Predict the Presence but Not the Severity of Obstructive Sleep Apnoea. A Systematic Review and Meta‐Analysis of the Literature

**DOI:** 10.1111/joor.13949

**Published:** 2025-02-14

**Authors:** Chee Weng Yong, Bernadette Quah, Nicole Li Shuen Kong, Juliana Tereza Colpani, Raymond Chung Wen Wong

**Affiliations:** ^1^ Faculty of Dentistry, National University of Singapore Singapore

**Keywords:** exostoses, obstructive sleep apnoea, torus mandibularis, torus palatinus

## Abstract

**Background:**

Oral torus assessment is recommended as a part of routine craniofacial examination in patients with obstructive sleep apnoea (OSA). However, there are conflicting studies on whether oral torus is associated with OSA and whether it affects OSA therapy.

**Objective:**

This study aimed to systematically review the effects of mandibular torus on OSA and its treatment.

**Methods:**

The PubMed, Embase and Cochrane Library databases were searched up to 15 July 2024. Studies that included patients with oral torus and examined the diagnosis and severity of OSA (Apnoea–Hypopnea Index [AHI], oxygen saturation, blood pressure and patient‐reported outcomes), and studies that examined the effectiveness of OSA treatment in patients with oral torus were included. PRISMA guidelines were followed for data extraction.

**Results:**

Eleven studies with 1372 patients were included in the study. Patients with mandibular torus were found to have a relative risk of 1.9 (95% CI = 0.9; 4.1) for OSA. The pooled mean difference in AHI between patients with and without mandibular torus was 1.6 (95% CI = −5.3; 8.6). Large mandibular torus was found to be associated with mild and moderate OSA but not with severe OSA. A greater reduction in AHI after mandibular advancement device or soft‐tissue OSA surgery can be achieved in patients with torus. However, the difference was not significant when compared to patients without it.

**Conclusion:**

Patients with mandibular torus are more likely to have OSA. Larger mandibular torus may be associated with mild or moderate OSA but not severe OSA. Mandibular torus does not impede OSA treatment.

## Graphical abstract

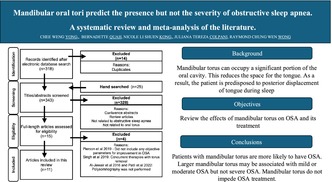

## 1 Introduction

Obstructive sleep apnoea (OSA) is a sleep‐related breathing disorder that involves a decrease or complete cessation of airflow, despite ongoing efforts to breathe. These patients may present with symptoms of sleepiness and daytime somnolence [1]. OSA has been linked to several health problems, including coronary artery disease, hypertension and diabetes mellitus [2]. Owing to the health implications of OSA, it is paramount to prevent or treat this disease.

When evaluating a patient with suspected OSA, it is common to conduct a craniofacial examination to identify anatomical conditions that may potentially reduce the patency of the upper airway or worsen the collapsibility of the upper airway structures [3]. A complete craniofacial examination typically also includes an examination of the oral cavity, where the clinician can assess pertinent structures such as the tongue, soft palate and oral torus.

Oral tori are benign bony outgrowths of the maxilla and mandible: a torus palatinus protrudes from the palatal surface of the maxilla, whereas a torus mandibularis protrudes from the lingual surface of the mandible. Multiple risk factors have been associated with the development or growth of these tori, including bruxism, hypertension and hyperthyroidism [4]. The prevalence of torus varies among different studies, from as low as < 1% to as high as > 60% [5]. Apart from the great variability in its prevalence, tori also differ greatly in their shape and size [6]. Due to their location and size, it is unsurprising that several reports have implicated the presence of oral torus as a cause of OSA.

It is common for guidelines to include oral torus evaluation as part of the examination [7, 8], although their specific implications are often not explained. The recommendations for assessing torus would typically imply that it would affect the prevalence, severity or treatment of OSA. There are postulations that the mandibular oral torus may potentially compete with the tongue for space within the oral cavity, leading to the displacement of the tongue and its associated soft tissues [9]. This in turn narrows the airway and increases the likelihood of OSA. Following this chain of thought, a larger torus could possibly lead to a greater degree of displacement, thus increasing the severity of OSA [9].

The presence of mandibular oral torus has also been theorised to affect OSA treatment. In patients who are not compliant with continuous positive airway pressure (CPAP) therapy, a mandibular advancement device (MAD) may be prescribed. These devices wrap around the alveolus for retention. Therefore, it is plausible that the fit and comfort of the MAD may be reduced and affect its efficacy [10]. Other authors have proposed that the presence of tori may also influence the effectiveness of surgical treatment of OSA and that the torus should be removed as part of the treatment plan [11, 12].

Conversely, there was also opposition to the negative effects of mandibular torus on OSA. In fact, some authors suggest that torus may even have a protective effect and positively influence treatment outcome [13, 14]. Given the polarised views of the role of mandibular oral tori in OSA, this systematic review aimed to investigate the relationship between mandibular oral tori and the presence and severity of OSA, as well as its effects on OSA treatment.

## 2 Materials and Methods

**FIGURE 1:**
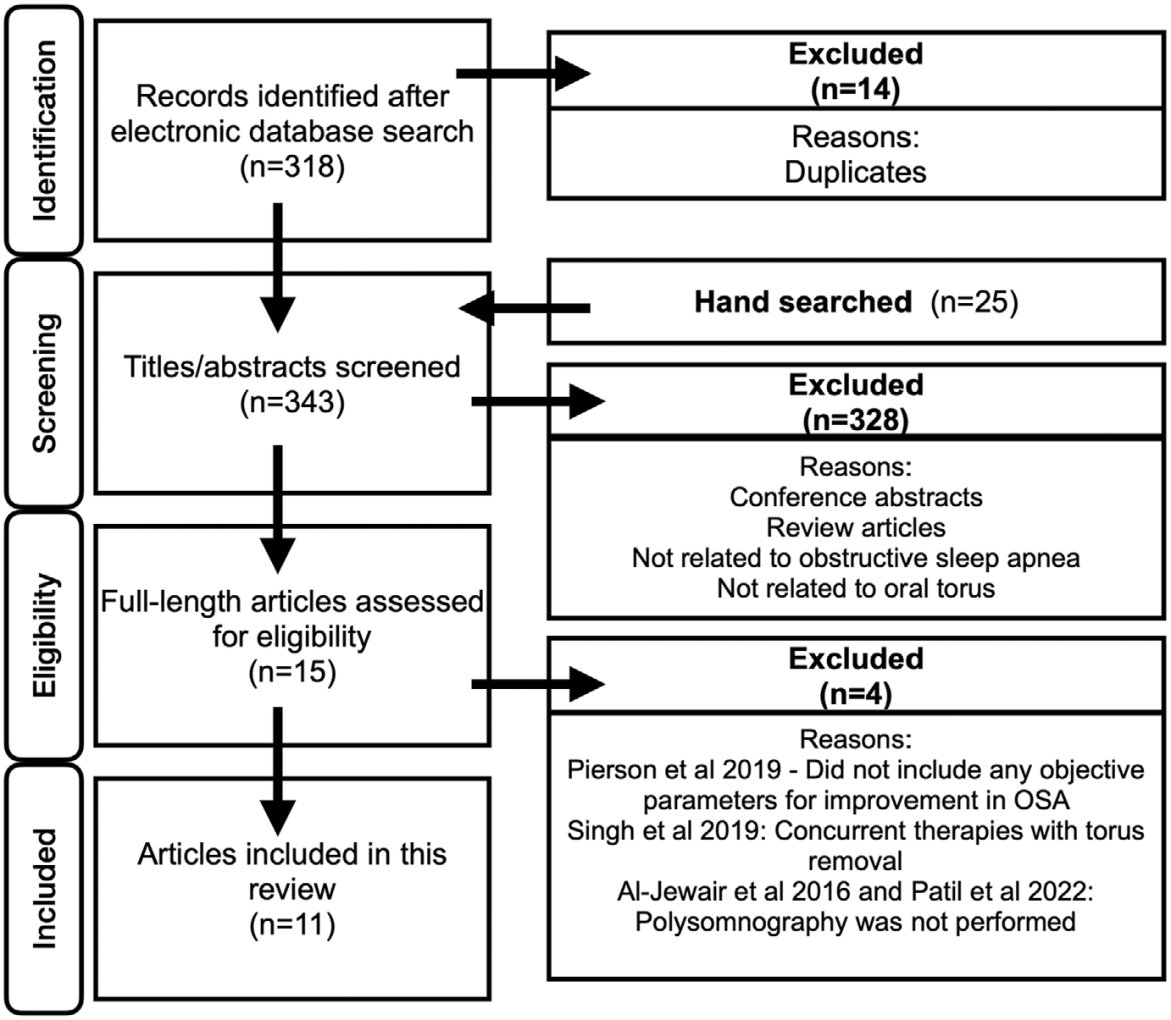
PRISMA flowchart.

This systematic review and meta‐analysis was conducted in accordance with the Preferred Reporting Items for Systematic Reviews and Meta‐analyses (PRISMA) statement for reporting systematic reviews (Figure 1) [15]. This study was registered with PROSPERO (CRD42024573821). The PubMed, Embase and Cochrane Library databases were searched in this study. Ethics approval was not required for this study. The following PEO process was used:

**Table:** 

Population	Patients with obstructive sleep apnoea
Exposure	Presence of oral tori
Outcome 1	Effects on OSA: Apnoea–Hypopnea Index (AHI), presence of OSA and severity of OSA
Outcome 2	Effects on OSA treatment outcome: AHI, oxygen saturation and patient‐reported outcome measures

### 2.1 Search Strategy

The terms used in the searches included torus, tori, exostosis, sleep apnoea syndromes, sleep apnoea, sleep apnoea, sleep disorder, sleep disordered breathing, appliance, device, splint, positional therapy, myofunctional, positive airway pressure, CPAP, PAP, nasal surgery, palatal surgery, palatoplasty, pharyngoplasty, palatopharyngoplasty, uvulopalatopharyngoplasty, hyoid suspension, hyoid myotomy, tongue surgery, tongue base surgery, tongue reduction, tonsillectomy, adenoidectomy, genioplasty, genial tubercle advancement, genioglossus advancement, maxillomandibular advancement, telegnathic surgery, mandibular advancement, tracheostomy, multilevel surgery, upper airway stimulation, hypoglossal nerve stimulation, sleep surgery and maxillary expansion (Annex A.1). The references of the selected articles were manually searched for additional studies to be included in the review.

### 2.2 Study Selection

A systematic search and study selection was independently conducted by two authors (CWY and BQ). Articles were screened using their titles and abstracts. The full text of the selected articles was then evaluated. In the event of disagreement, a third author's opinion was included (RCWW).

For the first outcome (effect on OSA), the inclusion criteria were as follows: (1) observational studies, non‐randomised and randomised controlled trials; (2) studies that only included patients with OSA or those that compared patients with and without OSA; (3) studies that compared patients with and without oral tori; (4) diagnosis of OSA was confirmed with polysomnography and (5) outcomes related to OSA, such as AHI or oxygen saturation, were recorded. The following studies were excluded: (1) studies not conducted on patients with OSA; (2) studies with no groups or comparisons made between patients with and without tori and (3) studies in which diagnosis of OSA was not determined by polysomnography.

For the second outcome (effect on OSA treatment), the inclusion criteria were as follows: (1) case reports, case series, observational studies, and non‐randomised and randomised controlled trials; (2) treatment was rendered to patients with OSA; (3) studies that included patients with torus; (4) the diagnosis of OSA was confirmed with polysomnography and (5) outcomes related to OSA, such as AHI or oxygen saturation, were recorded. The following studies were excluded: (1) studies not involving patients with OSA, (2) treatment outcomes not evaluated post‐operatively with polysomnography and (3) multiple therapies performed concurrently with torus removal, where the outcome may be attributed to these other therapies apart from torus removal.

### 2.3 Data Extraction

The following data were independently extracted by two authors (CWY and BQ): study design, demographic data, presence of tori, dimensions of the tori, treatment performed and OSA assessment outcomes. The author, year of publication, sample size, age, sex and body mass index (BMI) of the patients were collected as demographic data. For the treatment, details on the type of treatment and outcome measures were collected, including AHI (events/h), Respiratory Disturbance Index (RDI, events/h), minimal oxygen saturation (%), Oxygen Desaturation Index (ODI, average number of desaturation episodes/h), mean oxygen saturation (%), saturation < 90% (% of the time), lowest oxygen saturation (%) and patient‐reported outcomes.

### 2.4 Risk of Bias

The risk of bias of the studies was assessed using the Newcastle–Ottawa Quality Assessment Form and Joanna Briggs Institute (JBI) checklist for case reports [16, 17]. The assessments were performed independently by two authors (CWY and BQ). In the event of disagreement, the opinion of a third author (RWCW) was sought.

### 2.5 Statistical Analysis

Meta‐analyses were conducted to estimate the pooled change scores for the outcomes of interest (Meta Package, R Version 4.3.1). The mean, standard deviation (SD) and correlation coefficient of these outcomes, both before and after treatment, were extracted to estimate mean and SD changes. For studies in which the correlation coefficient before and after treatment was not explicitly provided, the value was imputed. Subsequently, meta‐analyses were conducted to estimate the pooled mean change score, with a confidence interval of 95%. Heterogeneity across the studies was assessed using the *I* ^2^ index. Random effects models were employed for effect size pooling in instances where *I* ^2^ was ≥ 75%, indicating substantial heterogeneity.

## 3 Results

The systematic review process is illustrated in Figure 1. A database search was conducted on 15 July 2024, retrieving a total of 318 articles (PubMed, *n* = 190; Embase, *n* = 111; Cochrane Library, *n* = 17). After removing the duplicates (*n* = 14), 42 articles were retrieved from manual searches (hand searched). Fifteen full‐length articles were evaluated and 11 were included in the review [9, 11, 12, 13, 14, 18, 19, 20, 21, 22, 23]. The inter‐rater agreement between the two authors (CWY and BQ) was *k* = 0.86, suggesting a high level of agreement between the two authors.

The included studies were published between 2010 and 2022. Of these, two were prospective, five were retrospective and the remaining four were case reports. The studies were conducted in Spain (1/11), Korea (2/11), Sweden (1/11), Thailand (3/11), Japan (2/11) and the United States (2/11). This systematic review included 1372 patients (4/1372 from case reports). The patients were predominantly male, with ages ranging from 30 to 60 years. The mean BMI was mostly within the overweight range (≥ 23 kg/m^2^). The details are summarised in Tables 1 and 2.

**TABLE 1:** Demographics of patients included in the assessment of the effects of torus on the presence and severity of OSA.

Author (year)	Study type	Country	Groups	Age (SD), years	M:F	BMI (SD), kg/m^2^	AHI (SD), event/h	SSD in AHI	Presence or severity of OSA	Torus assessment
Diaz de Teran et al. (2022) [13]	Prospective	Spain	Torus (*n* = 34/103)	45.15 (8.5)	30:4	BMI ≥ 30:6	28.7 (13.4)	No SSD, *p* = 0.250	All patients have OSA (*n* = 103/103)	Only mandible. Not graded; determined by clinical examination
			No torus (*n* = 69/103)	46.6 (9.5)	62:7	BMI ≥ 30:24	33.6 (17.4)			
Ahn et al. (2019) [12]	Retros pective	Korea	Torus (*n* = 94/232)	44.6 (14.3)	74:20	22.7 (1.5)	25.1 (18.4)	Yes, *p* = 0.006	No OSA: 14/38, mild OSA: 16/59, moderate OSA: 26/67, severe OSA: 38/68	Only mandible. Not graded; determined by clinical examination and computed tomography
			No torus (*n* = 138/232)	44.8 (15.3)	103:35	23.0 (3.2)	18.8 (14.9)		No OSA: 24/38, mild OSA: 43/59, moderate OSA: 41/67, severe OSA: 30/68	
Ahn et al. (2021) [11]	Retros pective	Korea	Torus (*n* = 35/104)	40.2 (12.0)	33:2	25.2 (3.4)	45.2 (19.9)	N/A	All patients have OSA (*n* = 104/104)	Only mandible. Not graded; determined by clinical examination and computed tomography
			No torus (*n* = 69/104)	44.9 (12.0)	57:12	25.7 (3.2)	42.1 (22.2)			
Palm et al. (2014) [14]	Retrospective	Sweden	Torus (*n* = 163/600)	51.7 (9.0)	523:77	27.8 (3.8)	133 with RDI > 4	N/A	No OSA: 42/138, mild OSA: 69/233, moderate OSA: 46/159, severe OSA: 16/70	Only mandible. Thickness, height and length of torus measured with a digital sliding calliper
			No torus (*n* = 437/600)				329 with RDI > 4			
Ruangsri et al. (2016) [9]	Prospective	Thailand	Large torus (*n* = 6/156)	NA	NA	NA	NA	NA	OSA: 5/6	Only mandible. Classified according to Agbaje et al. and Reichart et al.
			No large torus (*n* = 150/156)	NA	NA	NA	NA		OSA: 73/150	
Ruangsri (2020) [18]	Retrospective	Thailand	Torus (*n* = 7/131)	NA	NA	NA	NA	NA	No OSA: 111/131, mild OSA: 13, moderate to severe OSA: 7	Both torus palatinus and mandibularis. Not graded; determined by clinical examination
			No torus (*n* = 124/131)	NA	NA	NA	NA			
Pavarangkul et al. (2016) [19]	Retrospective	Thailand	Torus (*n* = 15/124) or (*n* = 15/42 OSA Px)	34.0 (19.3) or 59.5 (9.8) in OSA Px	75:48 or 27:15 in OSA Px	BMI > 25:38/124 33/42 in OSA Px	NA	NA	OSA: (*n* = 15/15)	11 and 4 Px have torus palatinus and mandibularis, respectively Not graded; determined by clinical examination
			No torus (*n* = 109/124) or (*n* = 27/42 OSA Px)						OSA: (*n* = 27/109)	

Abbreviations: AHI = Apnoea–Hypopnea Index, BMI = body mass index, M:F = male:female, *n* = sample size, OSA = obstructive sleep apnoea, Px: patients, SD = standard deviation, SSD = statistically significant difference.

**TABLE 2:** Pre‐ and post‐treatment parameters.

Author (year)	Study type	Country	Group/Tx	Pre‐ and post Tx AHI (event/h)	Pre‐ and post Tx ODI (event/h)	Pre‐ and post Tx Sat < 90% (% of time) or lowest Sat (%)	Pre‐ and post Tx mean Sat (%)	Pre‐ and Post Tx PROMs
Diaz de Teran et al. (2022) [13]	Retrospective	Korea	Torus/MAD (*n* = 34/103)	28.7 (13.4) to 7.1 (6.2), ***p*** **< 0.001**	22.1 (17.5) to 7.2 (7.6), ***p* < 0.001**	Sat ≤ 90% 6.5 (9.3) to 2.1 (3.7), ***p* = 0.024**	94.0 (1.6) to 94.0 (1.6), *p* = 0.312	NA
			No torus/MAD (*n* = 69/103)	33.6 (17.4) to 13.6 (9.9), ***p* < 0.001**	27.9 (19.5) to 11.7 (9.7), ***p* < 0.001**	Sat ≤ 90% 8.5 (14.6) to 5.8 (12.3), ***p* = 0.228**	92.1 (9.7) to 93.8 (1.6), *p* = 0.148	
Ahn et al. (2021) [11]	Retrospective	Korea	Torus/palatal surgery ± tongue base resection (*n* = 35/104)	45.2 (19.9) to 22.5 (13.5), ***p* < 0.001**	35.9 (21.6) to 27.5 (38.1), *p* = 0.274	Lowest Sat 79.2 (8.8) to 83.6 (6.2), ***p* = 0.019**	94.8 (1.5) to 95.5 (1.2), ***p* = 0.035**	ESS 8.5 (4.3) to 7.2 (3.9), *p* = 0.222 PSQI 7.9 (3.5) to 5.8 (2.0), ***p* = 0.009**
			No torus/palatal surgery ± tongue base resection (*n* = 69/104)	42.1 (22.2) to 23.9 (21.4), ***p* < 0.001**	35.3 (22.8) to 24.6 (37.1), *p* = 0.051	Lowest Sat 79.8 (8.2) to 83.9 (7.9), ***p* = 0.003**	94.4 (2.3) to 95.3 (2.5), ***p* = 0.045**	ESS 9.3 (5.0) to 7.9 (4.8) *p* = 0.099 PSQI 8.5 (4.5) to 6.2 (3.1), ***p* = 0.001**
Sato et al. (2018) [20]	Case report (47 F)	Japan	Mandibular torus removal	RDI 5 to 4.9	8.2 to 6.5	Sat ≤ 90% 8.23 to 2.08 Lowest Sat 32% to 76%	NA	ESS 6 to 4
McLeod et al. (2017) [21]	Case report (57 M)	USA	Mandibular torus removal	RDI 5 to 3.6	NA	Lowest Sat 88.6% to 88.6%	NA	NA
Singh et al. (2012) [22]	Case report (47 F)	USA	Mandibular torus removal	21.4 to 18.2	12.6 to 8.7	NA	NA	NA
Matsushita et al. (2010) [23]	Case report (59 M)	Japan	Mandibular torus removal	10.9 to 4.9 (1 week) to 14.5 (6 months)	9.7 to 12.7	NA	NA	ESS 15 to 6

*Note:* Palm et al.'s study is not included in the table as detailed information on torus and non‐torus subgroups on MAD treatment was not available; bold values, extracted from the relevant publication showing the confidence levels.

Abbreviations: AHI = Apnoea–Hypopnea Index, ESS = Epworth Sleepiness Scale, F = female, M = male, *n* = sample size, OSA: obstructive sleep apnoea, PSQI = Pittsburgh sleep Quality Index, Px: patients, RDI = Respiratory Disturbance Index, Sat = oxygen saturation.

Three hundred and fifty‐eight patients (26.1%) had oral tori. Nine of the studies investigated the presence of torus mandibularis only, whereas two studies investigated both torus palatinus and mandibularis [18, 19]. Most of the torus investigated in this study was located in the mandible. Only two studies evaluated the size of the torus mandibularis. Ruangsri et al. stratified the torus mandibularis using classification systems from Agbaie et al. and Reichart et al. [9], whereas Palm et al. measured the thickness, height and length of the torus using a digital sliding calliper [14].

### 3.1 Risk of Bias

The Newcastle–Ottawa Quality Assessment form and JBI checklists were used to assess seven primary studies and four case reports, respectively. Of the seven primary studies, five were evaluated to be of good quality, whereas the remaining two were deemed to be of fair quality. All four case reports were assessed as adequate for inclusion (Annex A.2).

### 3.2 Relationship of Mandibular Tori to OSA

**FIGURE 2:**
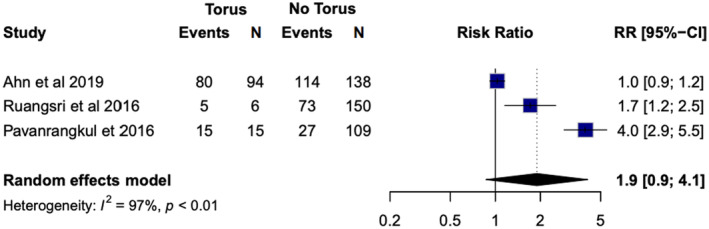
Meta‐analysis of the risk of OSA in patients with and without tori.

Seven studies were included in the analysis of the relationship between mandibular tori and OSA (the four case reports were excluded) [9, 11, 12, 13, 14, 18, 19]. The patient demographics are summarised in Table 1. For the evaluation of this outcome, 1368 patients were included (excluding four patients from case reports), of which 943 (68.9%) were diagnosed with OSA and 353 (25.8%) had oral tori. Three heterogeneous studies (*I* ^2^ = 97%, *p* < 0.01) distinctly reported the absence or presence of OSA in patients with mandibular torus, instead of just reporting the mean AHI or RDI. In these studies [9, 12, 18], OSA was observed in 82.5% of the patients with mandibular tori (208/252) and 68.7% of patients without mandibular tori (470/684). This finding showed that a greater proportion of patients with mandibular torus have OSA. The meta‐analysis showed a relative risk of 1.9 (95% CI = 0.9; 4.1) for patients with tori (Figure 2).

**FIGURE 3:**
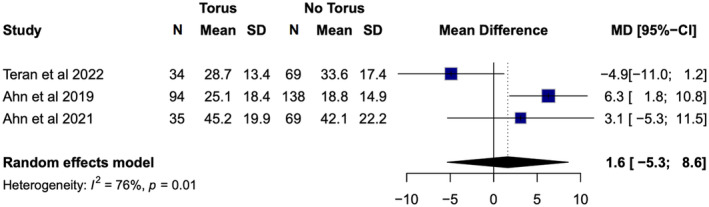
Meta‐analysis of mean AHI in patients with and without tori.

Three heterogeneous studies (*I* ^2^ = 76%, *p* = 0.01) evaluated AHI in relation to the presence of tori [11, 12, 13]. Patients with tori had a mean AHI of 30.1 ± 19.4, whereas those without tori had a mean AHI of 28.3 ± 20.2. The corresponding mean severity of the OSA was severe and moderate, respectively. Although the mean AHI resulted in different OSA severities, the difference was small. Meta‐analyses found a slightly higher AHI in patients with tori, with a pooled mean difference in AHI of 1.6 (95% CI = ‐5.3; 8.6) (Figure 3). Diaz de Teran et al. reported no statistically significant difference in the AHI between patients with and without tori (*p* = 0.250), whereas the opposite was reported by Ahn et al. (*p* = 0.006) [12, 13]. However, it is important to know that the patients included in Ahn et al.'s study were better matched for their demographics (especially BMI) as compared to the patients included in the study by Diaz de Teran et al. [2, 13]. A similar contrast can be seen in the relationship between mandibular tori and OSA severity. Palm et al. reported no statistically significant difference in the presence of mandibular tori between patients of different OSA severity (*p* = 0.562), whereas Ahn et al. [12] identified that patients with mandibular tori are more likely to have severe OSA, using both AHI (*p* = 0.007) and RDI (*p* = 0.034).

Although Palm et al. did not find statistically significant differences in the presence of mandibular tori in relation to the severity of OSA, the authors observed smaller mean torus heights in patients with severe OSA (5.1 ± 1.4 mm) compared to patients with mild OSA (6.5 ± 1.8 mm, *p* = 0.031) [14]. Although the mean torus height in severe OSA patients was also smaller than in patients with moderate OSA, the difference was not statistically significant (6.4 ± 1.8 mm, *p* = 0.057). The width and length of the mandibular tori were also noted to be lower in patients with severe OSA (*p* = 0.003 and *p* < 0.001, respectively). The height, width and length of the tori in patients with severe OSA were also found to be similar in size to those in snorers without OSA. Analysis from univariate logistic regression suggested that a torus thickness, height and length of at least 2.9 mm (OR 4.57, *p* = 0.012), 6.2 mm (OR 2.79, *p* = 0.020) and 17 mm (OR 3.91, *p* = 0.024), respectively, were related to an RDI of < 30. When matched for age, BMI and sex, a torus thickness of at least 2.9 mm was correlated with an RDI of < 30 (OR 4.7, *p* = 0.01). These findings showed that patients with mild or moderate OSA tended to have a larger torus, whereas patients with severe OSA tend to have a smaller torus. This finding is somewhat echoed by Ruangsri et al. [9]. The authors reported that a large mandibular torus (> 2 cm on both sides of the mandible) was slightly more common in patients with OSA (6.4% in OSA patients, 1.3% in controls, *p* = 0.360). However, multivariate logistic regression analysis identified a large mandibular torus (> 2 cm on both sides) as one of the factors that predicted OSA.

### 3.3 Relationship Between Mandibular Oral Tori and OSA Treatment

Seven studies (including four case reports) were included in the analysis of the relationship of mandibular oral tori to OSA treatment [11, 13, 14, 20, 21, 22, 23]. These studies included two that investigated the effects of oral tori on MAD therapy, one on soft‐tissue surgery and four on mandibular torus removal. Diaz de Teran et al. compared the outcomes of MAD treatment between patients with and without mandibular torus (34 and 69 patients, respectively). The authors found that MAD therapy was effective in both groups of patients in terms of reduction of AHI, ODI and duration with oxygen saturation < 90% (all improvements were statistically significant) [13]. However, the presence of mandibular tori was associated with an odds ratio of 3.54 (*p* = 0.007) for the effectiveness of MAD therapy. Palm et al. evaluated 217 patients with RDI ≥ 10 before MAD treatment [14]. The authors observed that complete treatment success (RDI < 5) was more common in patients with tori thicker than 2.9 mm. When adjusted for other variables, the odds ratio of complete treatment success in patients with thick tori was 2.5 (*p* = 0.02). Both studies suggested that the presence of mandibular tori does not reduce the effectiveness of MAD therapy and may even improve outcomes.

Ahn et al. investigated the influence of the presence of mandibular torus on the outcome of patients who underwent palatal surgery (with or without tongue base resection) [11]. They included 35 and 69 patients with and without a mandibular torus, respectively. Although the AHI reduction was greater in the mandibular torus group, it was not statistically significant. Regardless, the authors noted that soft‐tissue surgeries were effective for both groups of patients, measured by a reduction in AHI and an increase in the lowest oxygen saturation, mean oxygen saturation and patient‐reported outcomes (i.e., Epworth Sleepiness Scale [ESS] and Pittsburgh Sleep Quality Index [PSQI]).

Four case reports have described the effects of mandibular torus removal on OSA [20, 21, 22, 23]. Three studies showed modest changes in AHI or RDI (average of −1.57), whereas Matsushita et al. showed an increase in AHI (3.6) after a follow‐up period of 6 months. Although the AHI worsened, the patient maintained a significant reduction in the ESS score. The pre‐ and post‐treatment parameters are summarised in Table 2.

### 3.4 Hypotheses Proposed in the Included Studies

The studies were conflicted in their views of the role of the torus in OSA pathogenesis. Diaz de Teran et al. observed that patients with greater hyoid retroposition were more likely to have mandibular tori and that these patients tended towards a better treatment response. They proposed that mandibular tori were the result of a compensatory mechanism [13]. According to their hypothesis, patients at risk of OSA who have a distally positioned hyoid would require greater traction on the genioglossus muscle. Due to the increased genioglossus muscular pull on its bone attachment, the mandibular torus hypertrophies. Simultaneously, the strengthened genioglossus allows for more favourable soft‐tissue traction during MAD therapy. Palm et al. proposed a similar theory as to why patients with larger tori tended to have milder OSA [14]. The authors suggest that these patients have greater upper airway dilator activation, protecting them from increased resistance of the airway. The mandibular torus enlarges as a result of increased muscular activity. Along similar lines of strengthened muscle activity, Palm et al. suggested that patients with a mandibular torus have better muscular responses during MAD therapy. In addition, the authors proposed that a large mandibular torus can help hold the tongue upward and away from the pharynx, thus reducing OSA.

In contrast, Ahn et al. [11, 12] disagreed with these hypotheses. They proposed that the mandibular torus occupies space in the mandible, pushing the tongue posteriorly, leading to upper airway obstruction. However, they did not suggest how the presence of the mandibular torus resulted in a better response to palatal surgery (with or without tongue base reduction), as observed in their study. The idea of the torus reducing oral cavity volume, leading to OSA, was also echoed by Sato et al., McLeod et al., Singh et al. and Matsushita et al. [20, 21, 22, 23]. These authors also suggested that torus removal will increase the oral cavity volume, thus ameliorating the posterior displacement of the tongue and reducing OSA.

## 4 Discussion

This study sought to uncover evidence on the effects of mandibular oral tori on OSA and its treatment. The results of this investigation confirmed that patients with mandibular torus were more likely to have OSA. It was also observed that patients with larger mandibular torus may have a higher chance of having OSA compared to patients with no torus or with a smaller torus. This was correlated by several studies [9, 11, 12, 13, 14, 18, 19]. Interestingly, the findings of this study also revealed that the size of the mandibular torus is not proportional to OSA severity. In fact, patients with severe OSA were more likely to have a smaller torus than patients with mild or moderate OSA.

The authors of the included studies have proposed opposing opinions regarding the role of the mandibular torus in OSA. One group of the authors believed that the oral torus displaces the tongue and predisposes the latter to obstruct the upper airway [11, 12, 20, 21, 22, 23]. This perception is plausible because the proportion of soft‐tissue volume to oral cavity volume has been cited as a potential risk factor for OSA. Takeuchi et al. compared the oral cavity volume in both deceased and live patients, with and without OSA, using computed tomography [24]. The authors found that in patients who had OSA or died due to OSA, the oral soft‐tissue volume was significantly larger, whereas the oral air space volume was significantly smaller than that of patients without OSA [24]. Shi et al. compared the upper airway morphology between Dutch and Chinese adults with OSA [25]. The authors found that while Chinese patients had a significantly smaller maxillomandibular enclosure area, there was no significant difference in the ratio of the tongue area to oral cavity volume between the two groups of patients [25]. These studies highlighted the importance of a balance between the oral tissue volume and the oral cavity space. These findings may support the notion that the mandibular oral torus reduces oral cavity volume and increases the odds of OSA. However, this does not explain why patients with severe OSA tend to have smaller toruses.

The other group of authors suggested that mandibular torus does not pose a risk for OSA [13, 14]. Instead, the authors proposed that the mandibular torus gave hints of a compensatory mechanism. The mandibular torus could have increased because of increased activity of the tongue muscles. With stronger muscular activity, the tongue is less likely to be displaced during sleep. This could explain why patients with a larger mandibular torus tend not to ‘progress’ into the severe forms of OSA. However, further investigations are necessary to explain why the increased tongue muscle pulls increase the mandibular torus size but not any other attachments, that is, genial tubercle. In addition, it has been suggested that the mandibular torus may lift the tongue upward towards the palate. This places it in a more favourable position, which is less likely to lead to upper airway obstruction [26]. The same reasons were used to explain why MAD treatment and palatal surgery were more effective in patients with torus [11, 12, 13, 14]. Although this view also seems logical, it does not explain why patients with oral torus seemed to be more likely to have OSA and why removal of the mandibular torus can lead to improvements in OSA.

Although the views of both groups of authors were logical, they are incomplete in explaining the association between OSA and oral mandibular torus. Perhaps, determining the relationship of OSA and oral mandibular torus may be an incorrect approach. It is understood that oral torus is associated with temporomandibular joint disorders (TMJD) and parafunctional occlusal activities [27, 28]. At the same time, it is also well appreciated that TMJD and parafunctional occlusal activities are also associated with OSA [29, 30]. Interestingly, several studies have also found that patients with mild or moderate OSA are more likely to have TMJD and parafunctional activities such as sleep bruxism than patients with severe OSA [31, 32]. Sleep bruxism has been proposed to protect against OSA by protruding the mandible and helping to regain upper airway patency [31, 32]. When OSA is severe, sleep bruxism no longer serves as an effective compensatory mechanism, and thus, other more effective mechanisms, such as changes to respiratory efforts, are employed [31, 32]. Mandibular oral torus may thus be present as a result of bruxism activities, and it may be that the latter was protective rather than the torus. This notion remains to be validated, and it has been challenged that OSA and sleep bruxism were concomitant diseases, rather than truly associated [33].

The findings of this study suggest that the removal of the mandibular torus may not be necessary to facilitate successful OSA treatment. In contrast, the presence of a mandibular torus may indicate that the patient is more likely to have a good outcome after treatment. While Palm et al. suggested that the positive effect could arise from upward lifting of the tongue by the mandibular torus, there is insufficient evidence to support it [14]. If this is true, mandibular torus removal in these patients may potentially hamper treatment outcomes. However, mandibular torus removal has also been demonstrated to reduce AHI or RDI, albeit in modest amounts, and from case reports only. Although not elicited from the included studies, the explanation for why patients with mandibular torus tend to have better outcomes may be related to the compensatory mechanism of sleep bruxism.

All the available studies focused on the mandibular torus and not the maxillary torus. This is surprising in light of the fact that in the recent decade, there has been a greater focus on maxillary expansion to create space for the tongue and that the inability to position the tongue against the hard palate has been associated with OSA [34, 35]. The maxillary torus shares an equally variable prevalence and morphology with the mandibular torus but has received less attention [5].

A key limitation of this systematic review was the absence of randomised controlled trials. Therefore, the results of this study cannot be used to develop meaningful guidelines. However, it serves as a reminder for clinicians to challenge their perspectives and to have deeper considerations during their assessment of patients with OSA. Apart from the lack of randomised controlled trials, there was a significant heterogeneity among the included studies. The patient groups differed in demographics, pre‐treatment AHI, treatment and assessment methods. This limited the potential of this meta‐analysis. Third, most studies did not record the size or morphology of the oral torus. Thus, it was not possible to analyse the effect of torus size on OSA across studies. Apart from a randomised controlled trial study model, future primary studies should assess the relationship between the triad of oral torus, sleep bruxism and OSA. Apart from volumetric changes, it would also be meaningful to investigate the positioning of the tongue in patients with torus. The tongue is mobile and can be deformed, and it is possible that there are factors, apart from absolute volumetric reduction in the oral cavity space, that influence the prevalence and treatment of OSA.

## 5 Conclusion

Patients with mandibular tori are more likely to have OSA, and larger tori are generally associated with its presence. While larger tori are more likely to be associated with OSA, patients with severe OSA tend to have smaller tori. The evidence is conflicted on whether tori are risk factors for OSA or if they are a protective effect against severe OSA. Regardless, removal of the tori may not be necessary for MAD therapy or soft‐tissue OSA surgery to be effective. Although tori removal can ameliorate OSA, the improvements are modest. More research is still required to determine the link between tori and OSA, particularly within the context of craniofacial patterns and functional dynamics.

## Author Contributions

C.W.Y.: Conceptualisation, methodology, formal analysis, writing – original draft, visualisation. B.Q: Methodology, formal analysis, writing – original draft. N.L.S.K: Conceptualisation, writing – review and editing. J.T.C: Writing – review and editing, validation. R.C.W.W: Conceptualisation, writing – reviewing and editing, supervision, resources.

## Ethics Statement

The protocol for this study was registered with PROSPERO (CRD42024573821).

## Conflicts of Interest

The authors declare no conflicts of interest.

## Data Availability

Data available on request from the authors.

## Appendix A

### Annex A—Search Strategy

Pubmed

**Table:**

#1	((torus) OR (tori) OR (exostosis)) AND ((Sleep apnea syndromes[Mesh]) OR (Sleep apnea) OR (Sleep Apnoea) OR (OSA) OR (Sleep disorder*) OR (sleep disordered breathing))
#2	((torus) OR (tori) OR (exostosis)) AND ((Sleep apnea syndromes[Mesh]) OR (Sleep apnea) OR (Sleep Apnoea) OR (OSA) OR (Sleep disorder*) OR (sleep disordered breathing)) AND ((Appliance) OR (Device) OR (Splint))
#3	((torus) OR (tori) OR (exostosis)) AND ((Positional therapy) OR (Myofunctional) OR (Positive airway pressure) OR (CPAP) OR (PAP))
#4	((torus) OR (tori) OR (exostosis)) AND ((nasal surgery) OR (Palatal surgery) OR(Palatoplasty) OR (Pharyngoplasty) OR (Palatopharyngoplasty) OR (uvulopalatopharyngoplasty) OR (hyoid suspension) OR (Hyoid myotomy) OR (tongue surgery) OR (tongue base surgery) OR (tongue reduction) OR (Tonsillectomy) OR (Adenoidectomy) OR (Genioplasty) OR (Genial tubercle advancement) OR (Genioglossus advancement) OR (Maxillomandibular advancement) OR (Telegnathic surgery) OR (Mandibular advancement) OR (tracheostomy) OR (multilevel surgery)OR (upper airway stimulation) OR (hypoglossal nerve stimulation) OR (Sleep surgery) OR (Maxillary expansion))

Embase

**Table:**

#1	(torus OR tori OR exostosis) AND (sleep AND apnea AND syndromes OR (sleep AND apnea) OR (sleep AND apnoea) OR osa OR (sleep AND disorder*) OR (sleep AND disordered AND breathing))
#2	(torus OR tori OR exostosis) AND (sleep AND apnea AND syndromes OR (sleep AND apnea) OR (sleep AND apnoea) OR osa OR (sleep AND disorder*) OR (sleep AND disordered AND breathing)) AND (appliance OR device OR splint)
#3	(torus OR tori OR exostosis) AND (positional AND therapy OR myofunctional OR (positive AND airway AND pressure) OR cpap OR pap)
#4	(torus OR tori OR exostosis) AND (Nasal surgery OR Palatal surgery OR Palatoplasty OR Pharyngoplasty OR Palatopharyngoplasty OR uvulopalatopharyngoplasty OR hyoid suspension OR Hyoid myotomy OR tongue surgery OR tongue base surgery OR tongue reduction OR Tonsillectomy OR Adenoidectomy OR Genioplasty OR Genial tubercle advancement OR Genioglossus advancement OR Maxillomandibular advancement OR Telegnathic surgery OR Mandibular advancement OR tracheostomy OR multilevel surgery OR upper airway stimulation OR hypoglossal nerve stimulation OR Sleep surgery OR Maxillary expansion)

Cochrane

**Table:**

#1	((torus) OR (tori) OR (exostosis)) AND ((Sleep apnea syndromes) OR (Sleep apnea) OR (Sleep Apnoea) OR (OSA) OR (Sleep disorder) OR (sleep disordered breathing))
#2	((torus) OR (tori) OR (exostosis)) AND ((Sleep apnea syndrome) OR (Sleep apnea) OR (Sleep Apnoea) OR (OSA) OR (Sleep disorder) OR (sleep disordered breathing)) AND ((Appliance) OR (Device) OR (Splint))
#3	((torus) OR (tori) OR (exostosis)) AND ((Positional therapy) OR (Myofunctional) OR (Positive airway pressure) OR (CPAP) OR (PAP))
#4	((torus) OR (tori) OR (exostosis)) AND ((nasal surgery) OR (Palatal surgery) OR(Palatoplasty) OR (Pharyngoplasty) OR (Palatopharyngoplasty) OR (uvulopalatopharyngoplasty) OR (hyoid suspension) OR (Hyoid myotomy) OR (tongue surgery) OR (tongue base surgery) OR (tongue reduction) OR (Tonsillectomy) OR (Adenoidectomy) OR (Genioplasty) OR (Genial tubercle advancement) OR (Genioglossus advancement) OR (Maxillomandibular advancement) OR (Telegnathic surgery) OR (Mandibular advancement) OR (tracheostomy) OR (multilevel surgery)OR (upper airway stimulation) OR (hypoglossal nerve stimulation) OR (Sleep surgery) OR (Maxillary expansion))

### Annex B

#### Newcastle–Ottawa Quality Assessment

**Table:**

	Diaz de Teran et al. (2022) [13]	Ahn et al. (2019) [12]	Ahn et al. (2021) [11]	Palm et al. (2014) [14]	Ruangsri et al. (2016) [9]	Ruangsri et al. (2020) [18]	Pavarangkul et al. (2016) [19]
Representativeness of the exposed cohort	^a^	^a^	^a^	^a^	^a^	^a^	^a^
Selection of the non‐exposed cohort	^a^	^a^	^a^	^a^	—	^a^	^a^
Ascertainment of exposure	^a^	^a^	^a^	^a^	^a^	^a^	^a^
Demonstration that outcome of interest was not present at start of study	—	—	—	—	—	—	—
Comparability of cohorts on the basis of the design or analysis	—	—	—	—	—	—	—
Assessment of outcome	^a^	^a^	^a^	^a^	—	—	^a^
Was follow‐up long enough for outcomes to occur	^a^	^a^	^a^	^a^	^a^	^a^	^a^
Adequacy of follow‐up of cohorts	^a^	^a^	^a^	^a^	^a^	^a^	^a^
Quality	Good	Good	Good	Good	Fair	Fair	Good

^a^ Fulfils one of the criteria listed.

#### Joanna Briggs Institute Checklist

**Table:**

	Sato et al. (2018) [20]	McLeod et al. (2017) [21]	Singh et al. (2012) [22]	Matsushita et al. (2010) [23]
Were patient's demographic characteristics clearly described?	Y	Y	Y	Y
Was the patient's history clearly described and presented as a timeline?	Y	Y	Y	Y
Was the current clinical condition of the patient on presentation clearly described?	Y	Y	Y	Y
Were diagnostic tests or assessment methods and the results clearly described?	Y	Y	Y	Y
Was the intervention(s) or treatment procedure(s) clearly described?	Y	Y	Y	Y
Was the post‐intervention clinical condition clearly described?	Y	Y	Y	Y
Were adverse events (harms) or unanticipated events identified and described?	N	N	N	N
Does the case report provide takeaway lessons?	Y	Y	Y	Y
Adequate for inclusion	Y	Y	Y	Y

Abbreviations: Y = yes, N = no.
